# Solving a methodological challenge in work stress evaluation with the Stress Assessment and Research Toolkit (StART): a study protocol

**DOI:** 10.1186/1745-6673-8-18

**Published:** 2013-06-22

**Authors:** Dina Guglielmi, Silvia Simbula, Michela Vignoli, Ilaria Bruni, Marco Depolo, Roberta Bonfiglioli, Maria Carla Tabanelli, Francesco Saverio Violante

**Affiliations:** 1 Department of Educational Sciences, University of Bologna, Via Filippo Re 6, Bologna 40126, Italy; 2 Department of Psychology, University of Milano Bicocca, Piazza dell’Ateneo Nuovo 1, Milano 20126, Italy; 3 Department of Psychology, University of Bologna, Viale Berti Pichat 5, Bologna 40127, Italy; 4 Occupational Medicine - Department of Medical and Surgical Sciences, University of Bologna, Via Pelagio Palagi 9, Bologna 40138, Italy

**Keywords:** Stress, Psychosocial risk evaluation, Mixed methods, Study protocol

## Abstract

**Background:**

Stress evaluation is a field of strong interest and challenging due to several methodological aspects in the evaluation process. The aim of this study is to propose a study protocol to test a new method (i.e., the Stress Assessment and Research Toolkit) to assess psychosocial risk factors at work.

**Design:**

This method addresses several methodological issues (e.g., subjective *vs*. objective, qualitative *vs* quantitative data) by assessing work-related stressors using different kinds of data: i) organisational archival data (organisational indicators sheet); ii) qualitative data (focus group); iii) worker perception (questionnaire); and iv) observational data (observational checklist) using mixed methods research. In addition, it allows positive and negative aspects of work to be considered conjointly, using an approach that considers at the same time job demands and job resources.

**Discussion:**

The integration of these sources of data can reduce the theoretical and methodological bias related to stress research in the work setting, allows researchers and professionals to obtain a reliable description of workers’ stress, providing a more articulate vision of psychosocial risks, and allows a large amount of data to be collected. Finally, the implementation of the method ensures in the long term a primary prevention for psychosocial risk management in that it aims to reduce or modify the intensity, frequency or duration of organisational demands.

## Background

Currently, work-related stress represents one of the biggest challenges for health and safety at work. Disorders associated with stress (fatigue, perceived stress, irritability, headaches, etc.) are second only to musculoskeletal disorders and affect 22% of European workers [1]. Furthermore, in the recently published Fifth European Working Conditions Survey, 59% of European workers report working at high speed and 37% report not being able to choose their method of work [2]. Notably, among psychosocial factors, organisational constraints (i.e., not having job-related information, having limited time and materials, or not having the necessary authority to be able to complete one’s tasks) have been shown to be strongly related to physical symptoms, such as backache, headache or sleep disturbances [3].

Work stress has been recognised as impacting heavily on the productivity and health costs of companies and countries: as studies of stress-related illness and mortality show, stress has a big effect on individual health and well-being as well as on organisational productivity [4,5]. In fact, recent findings confirm that productivity is jeopardised by the increase in absenteeism [6] and turnover rates [7].

Methodological improvement in detecting and measuring psychosocial risks is needed to add value to prevention plans and interventions, due to the fact that in the last few decades, the focus on psychosocial risk factors has become increasingly important because of their impact on health and well-being through the phenomenon of work-related stress [8].

Psychosocial risk factors refer to potential psychological, social and even physical damage to a worker due to the organisation and management of work or to job characteristics [9]. These factors could be operationalised by two key job characteristics, job demands and job resources, whose key roles are recognised in several models of work stress (e.g., [10-12]). Consideration of positive characteristics is of importance as it emphasises the role the positive aspects of the work activities have and how they allow workers to grow professionally.

Probst [13] recently claimed that the absence of theory is one of the main weaknesses in research on effective stress intervention. As Hargrove and colleagues [14] noted, at least a dozen theoretical models of organizational stress do exist: this means that a variety of non-identical approaches also exist, therefore it is important to explicit to which research paper is best to make reference. However, as Nixon and colleagues noted [3], theoretical models aimed at explaining the work-related stress process contain a huge amount of similar variables and elements (e.g., [15-17]). The underlying process posits a stimulus–response process in which job stressors lead to psychological or physical strain, and behavioural reactions, while a central role is played by individual appraisal of stressors. Emotional responses, such as anxiety and frustration, are often the most immediate psychological strain responses that are associated with physiological changes in the body [17]. Such a shared core set of variables/elements among different models does not imply that they are identical, but seems to confirm that in recent years no relevant modifications have been made to the theoretical approach towards work and organizational stress.

This paper will thus assume a shared set of variables (formed by the triad (a) stimulus–response; (b) individual appraisal shaping response, mainly by a comparison between demands and resources; and (c) psychological and physiological changes as a consequence of exposure to stressors) as the theoretical frame of reference, since its focus and aim is a methodological one. In other words, we want to present a potential methodological improvement in detecting psychosocial risks in work settings, assuming that our methodological approach is compatible with the shared basic set of components forming the most known and referred to existing models of work and organizational stress.

### Methodological features in work-related stress assessment

When dealing with psychosocial risks assessment there are several methodological aspects that have to be taken into account. In particular, three aspects are discussed: thresholds, measures and types of data.

#### Thresholds

Occupational hazards include exposure to chemicals, biological agents and allergens, as well as numerous physical factors, complex safety risks and many varied psychosocial risks [18]. In the case of environmental risk, the thresholds exposure to physical or chemical agents is relatively easy to define in respect to psychosocial risks where they depend essentially on the results of a cognitive evaluation [19]. The European Union clearly summarises such methodological difficulty in the definition of stress: “*stress is a state*, *which is accompanied by physical*, *psychological or social complaints or dysfunctions and which results from individuals feeling unable to bridge a gap with the requirements or expectations placed on them*. […]. *Moreover*, *different individuals can react differently to similar situations and the same individual can react differently to similar situations at different times of his*/*her life*” [20].

#### Types of measure (subjective/objective)

Most of the information available today on stress assessment instruments is derived from the use of subjective research tools e.g., [21] built on theoretical models [22,23] and scales of measurement based mainly on workers’ perceptions of their working conditions [24]. Investigating psychosocial risk factors by means of subjective tools only, is likely to produce measurement bias resulting from the personal interpretations of risk factors [25]. Indeed, responses may be distorted by response styles, the attribution process, personality characteristics or affective states [26], meanwhile, another drawback is related to the measurement of psychosocial variables and their outcomes, which can lead to common method variance [27]. On the other hand, objective approaches – such as observation or archival data – may reduce measurement bias, since they are independent from workers’ subjective perception. Additionally, observational measures may be affected by bias related to an observer’s interpretation, and rely heavily on the reliability of observation grids: the more job-specific they are, the more reliable they become, yet are less generalisable across jobs or work settings.

However, highly objective data does not offer *per se* more valid and reliable information: for instance, absences due to illness, performance measurements or accident records are incomplete indicators and they can in no way be treated as direct measures of psychosocial risks, since many other factors co-influence them. Among others, Kompier [26] argued that a multi-source approach may overcome the limitations mentioned above. Objective (i.e., archival data, observational data) and subjective (self-report questionnaires and focus groups) data can be used to reduce the measurement error typical of each kind of data-gathering tool.

#### Type of data (qualitative/quantitative)

Since researchers and practitioners agree that stressful conditions do not automatically lead to stress, it is important to rely on different typologies of data collection to increase the measure validity.

In fact, it is commonly agreed that quantitative and qualitative data provide different representations: to be specific, the former allows for easier comparisons and hypothesis testing, mainly by statistical tests, while the latter provide a deeper knowledge of the phenomenon under study. The usage of both typologies may lessen the biases that are specific to each one.

As in other fields of research, a mixed methods approach (for a review, cf. [28]) can respond to such issue. Mixed methods research is formally defined here as the combination of quantitative and qualitative research techniques, methods, approaches, concepts or languages into a single study. The use of methods should be predominantly influenced by substantive research questions, and not only by methodological and epistemological considerations. Like every method it has specific limitations as well as particular strengths; many scholars argue that qualitative and quantitative methods should be combined in order to compensate for their mutual and overlapping weaknesses [29]. It is thus necessary to try to locate these data collection methods within a solid theoretical framework as the approach presented is. As Ågerfalk [30] recently pointed out the number of reasons for choosing the discussion of why mixed methods research may be helpful, are numerous. Our approach uses *triangulation* (i.e., convergence and corroboration, which are made evident by different methods and designs), *complementarity* (i.e., results from one method clarify or integrate the results from another), and *development* (i.e., results from one method give information to improve research design involving another method).

### Aim

On the one hand, stress is a construct that has to be assessed both subjectively and objectively, on the other hand psychosocial risks in work settings have to be assessed in the most valid, reliable and feasible way.

Thus, the aim of this work is to develop a protocol study to propose a new methodology to assess psychosocial risk factors at work. The method is called the Stress Assessment and Research Toolkit (StART). The protocol presented uses a mixed methods approach to work-related stress assessment to overcome the methodological limitations presented above.

## Methods/design

### Participants

The stress assessment process cannot be strictly the same for every organisation, especially in the case of complex organisations with multiple roles. Thus the participants have to be divided into different groups according to the suggestions of the European Agency for Stress and Health at Work, which indicates that the first step to risk assessment is the identification of hazards and people at risk [31]. This way it is possible to form groups based on different work activities and stress risk exposures. In order to create adequate groups it is moreover necessary to involve a steering committee (described later) that can help to define the common risk among workers, defined by different work activities within the organisation.

Subsequently, it is necessary to draw a sample of workers representative of the organizational population, whose criteria for representativeness may vary, according to specific features of the organization researched; however the most important criteria (according to the existing literature in the field) are gender, age (and/or organizational seniority), organizational unit, job position. In some cases, where a complete coverage of different organizational units may be impossible (consider for example chain store organizations) it is possible to proceed with a clustering analysis using quantitative data regarding organizational local units (i.e. sales figures, worked hours, sales area and number of workers for every local unit).

### The method stages

**Figure 1:**
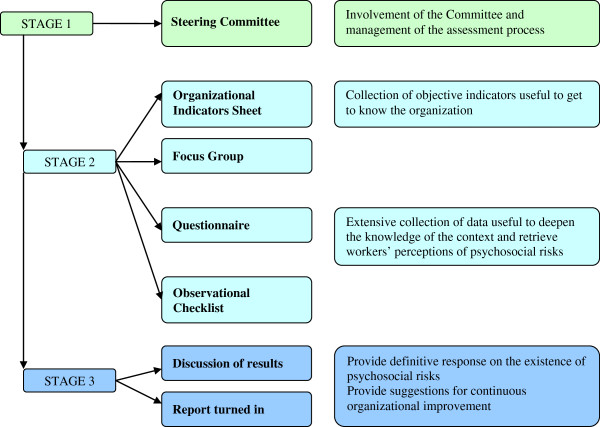
The assessment process in StART method.

The StART method encompasses several stages (Figure 1) that envisage the utilisation of different instruments specifically created to respond to the objectives of each stage.

#### Stage 1 – Involvement of the steering committee and management of the assessment process

Traditionally, the top-management involvement has been stressed as an important determinant of success [32,33]. The positive effects of management involvement include: enhanced organisational learning, stronger organisational commitment, higher job satisfaction, more adaptive core competencies, the development of competitive advantage and improved organisational performance [34,35].

The process begins with the identification of the roles necessary to provide information on the context (i.e., organisational indicators, development systems, etc.), and to manage and coordinate the assessment process. The organisational roles involved in the steering committee are the executives that could help the researchers to comprehend fully the data and manage the process. The roles usually involved are the human resources executive, who is responsible for safety, and the company physician.

In order to achieve the specific objectives of each stage, this phase endures all along the process.

#### Stage 2 – Collection of data

Stage 2 encompasses four sub-stages.

The first sub-stage aims to collect objective indicators – by means of the *Organisational Indicators Sheet* (OIS) – which are useful for getting to know the organisation. In some countries (e.g., Italy), the use of objective indicators of organisational functioning is advocated by national agencies [36]. The set of indicators is chosen among organisational archival data with the intent of collecting objective and numerable markers (e.g., sickness absence, injuries). Thus, they make reference mainly to: organisational structure, turnover, sickness absence, injuries, human resource practices and environmental risks. As Rugulies noted [37], archival data are useful for assessing quantitative and emotional demands at work.

The following sub-stages foresee the participation of workers. The goal is to collect data regarding individuals’ working conditions and individual perceptions using a focus group, questionnaire and observational checklist. The choice of appropriate instrument at this stage is determined by the type of information to be detected or the peculiarities of the context.

Data collected during the sub-stages are essential for deepening knowledge of the context and interpreting the results at the end of the assessment process.

The tools used at this stage are described in the following.

The second sub-stage envisages the use of *focus group* technique. This qualitative technique may be used in a preliminary phase to address the most common issues, which derive from the lack of knowledge of the context (cf. [38]). The use of a focus group allows information to be retrieved on the stable characteristics of the organisation and workers’ shared knowledge. This methodology is useful because it provides information about the degree of consensus and disagreement. Furthermore, such an instrument allows for richer information because it helps to determine how much all data contribute to saturation for the focus groups (the so-called saturation within-group data [39]).

Groups are formed considering that enough diversity can stimulate discussion, while an appropriate level of homogeneity can facilitate comparison between groups [40].

Focus groups are designed and conducted following the instructions provided by the literature [38,41,42]. During the sessions, two members of the research group play two different roles, one as a facilitator and moderator of the group work, and the other as an external observer, who tracks the interactions between participants taking field notes. A semi-structured frame is established prior to the beginning of each session, indicating that the participants can intervene in a way that is as similar as possible to an informal conversation. In order to guide and reactivate the conversation when necessary, the interviewers can use a script with questions referring to psychosocial risks and characteristics of the work environment, covering all the relevant levels of analysis (including personal, interpersonal and organisational). The questioning route also takes into account relevant information obtained during the preparatory meetings with the steering committee.

**Figure 2:**
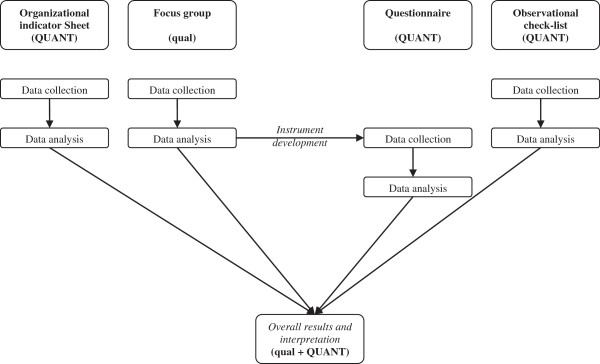
**Visual diagram of the procedures used in StART method.** The notation system is according to Creswell and Plano Clark [52]. The capital letters in QUANT refer to the greater weight assigned to the quantitative data, while qualitative data resulting from the focus group serve the purpose of guiding the choice of the questionnaire scales and are merged with the other results to assess conjointly the presence of psychosocial risks related to stress.

The focus group developed in the StART method includes questions on organisational psychosocial risks. Focus group results may also serve as a basis to guide the choice of the scales that constitute the questionnaire (see also Figure 2).

The next two sub-stages do not strictly follow a sequential path.

The third sub-stage is *questionnaire* administration. The questionnaire is the most utilised instrument in work-related stress research [37] because it is one of the most reliable tools for assessing workers’ perceptions of psychosocial risks. It collects quantitative data that allow a comparison between subjects or groups, and has moderate costs. Unlike other questionnaires used in order to perform the assessment of the psychosocial work environment (e.g., the Job Content Questionnaire, and Effort-Reward Imbalance), the questionnaire of the StART method is not stable in its composition: within a general stable framework, it is intentionally context-specific. According to Bakker and Demerouti [10], every work environment is considered to have its own specific risk factors associated with job stress. These factors can be classified into two general categories, which are job demands (e.g., workload, role conflict) and job resources (e.g., control, social support). For these reasons, the questionnaire encompasses two sections. The first (which is stable) investigates the factors more associated with work-related stress in literature, both job demands (e.g., workload, decision authority, work-family balance) and job resources (e.g., social support). Moreover, it includes personal outcomes (e.g., burnout, work engagement).

The second section strictly depends on the results of the focus groups, the data collected with the OIS and the information collected during the meetings with the steering committee.

The validity of such a questionnaire is supported by the selection of widely validated scales (e.g., for equity the scale validated [43]) or the validation of the entire questionnaire related to specific work environments, for instance the school context [44] and the hospital setting [45].

The fourth sub-stage concerns the *observational checklist* (OC). The main aim of the observation is to make an objective evaluation of psychosocial risk factors related to specific job positions. The focus of the observation is not the single physical person, but rather the typical tasks of a given organisational position. The objective is to identify job demands and events that may impede or interrupt the worker activities: potential stressors are considered a disturbance in the work regulation process, as they represent conditions that hinder the achievement of the objectives when there are no resources that can be used to cope with such obstacles [46].

Moreover, in order to overcome the potential bias related to an observer’s interpretation, it is important that the observers are therefore trained intensively. In particular there should be: a) a preliminary study of the observation methodology (including potential observation biases); b) an analysis of general informations about occupations (as well as a job analysis); c) at least two tutorial exercises observations with subsequent discussion of problems encountered; finally d) a full comprehension of dimensions of the observational checklist.

Indicators chosen to detect psychological risk factors depend on the results of the preliminary phase (information collected via OIS and focus groups) and the tasks performed by the specific position observed.

To date, few studies have tried to combine different sources of data [46,47]. One example is the ISTA method (Instrument for Stress-Oriented Task Analysis [25]), which starts from the concept of action regulation describing work from a psychological perspective as accomplished by goal-oriented action [48], and tries to match workers’ subjective perception with expert assessment through observation of the workers’ work environment. Nevertheless, in the ISTA method the subjectivity of the workers is replaced by the subjectivity of the observer, who has to answer to the same self-report rating scales previously administered to workers while observing them. In order to overcome this limit to objectivity, we developed an observational checklist, in which the variables were operationalised using observable aspects of the work activities. For instance, in the OC the variable “Task Variety” was measured via the observable number of different tasks performed in the time lag of observation, instead of asking the observer to give a rating rate on the same scale administered to the workers with the self-report questionnaire [49].

The instruments that collect quantitative data (questionnaire and OS) have already been validated. Two studies validated the questionnaire [44,45], others reported on the use of the OC [50], and on the integration between objective and subjective data [49].

The instruments illustrated above can, together, provide different information. However, the utilisation of all instruments is not mandatory. In fact, the implementation of the StART method could be flexible enough to allow different combinations of sub-stages (and then tools). This is due both to the organisational characteristics, such as the size and complexity of the organisational departments, and to the applicability of the instrument in the organizational context for issues relating to the privacy of the participants and ethics.

For instance, observation may not be feasible in a firm where organisational positions are mostly of a white-collar or managerial type, making the direct observation of specific tasks almost impossible. Another special condition may be the small size of the enterprise: in such a case, group discussions may replace questionnaires.

Given the importance attributed to the key role of job resources in the model, all instruments detect both negative and positive aspects of work. For instance, OIS reports the frequency of injuries and the yearly amount of training per employee.

#### Stage 3 – Provide a response on psychosocial risks

Preliminary results are discussed with the steering committee, which provides essential interpretation and helps to interpret potentially controversial results.

On the whole, the assessment process is conceptualised as a cycle. This strategy has already been suggested for the control of physical hazards, under the label of “Control Cycle”, which has been defined as “the systematic process by which hazards are identified, risks analysed and managed, and workers protected” [51]. This approach is nowadays recognised as the best professional practice in risk assessment [52].

Stage 3 brings us to the identification of psychosocial risk factors but it is not the end of the process. In fact, even if there is no evidence of potentially harmful psychosocial risks in the organisation, a set of suggestions for improvement are proposed so it can be monitored in the long term (e.g., if significant organisational changes occur). The monitoring of psychosocial factors and the implementation of improvement actions imply that the cycle may continue, beginning again from previous stages.

### Study design

We previously illustrated the different instruments and stages that constitute the StART framework. Certainly, using several data sources produced a vast and heterogeneous set of data, both qualitative and quantitative, which are difficult to integrate. Accordingly the study design proposed for the protocol is a mixed methods design [53], whose aim is to compare and contrast quantitative statistical research with qualitative findings or to validate or expand quantitative results with qualitative data.

The most common and well-known approach to mixing methods is the triangulation design [53]. The purpose of this design is “to obtain different but complementary data on the same topic” [54] to understand most effectively the research problem.

The intention in using this design is to bring together differing strengths and non-overlapping weaknesses of quantitative methods (large sample size, trends, generalisation) with those of qualitative methods (small N, details, in depth) [55].

This approach has several advantages: it describes the relationships between qualitative and quantitative findings and theoretical concepts in a study; it demonstrates how both qualitative and quantitative data can be integrated to improve the understanding of a particular phenomenon; and it can also be used to build on new theory [56].

As illustrated in Figure 2, in the StART method the integration between qualitative and quantitative data is carried out for two purposes: development and complementarity.

As regards *development*, the qualitative data (those with least weight) help to enhance the subsequent creation of the questionnaire (quantitative data with dominant weight).

Concerning *complementarity*, the main objective is to clarify or illustrate the results obtained with one method by also applying the other. In this case, the designs used are sequential, given that the qualitative part helps to evaluate and interpret the results obtained from the quantitative data [28].

Figure 2 presents how the different data are first collected, then analysed and, lastly, put together to allow for an integrated interpretation. As the intention is to test a method to assess work-related stress, the triangulation mixed-methods design combines both quantitative (OIS, questionnaire, OC) and qualitative (focus group) data. The results of every data analysis are merged in the final interpretation.

### Data analysis

Using mixed methods methodology a clear definition of variable measures is expected. Table 1 presents the risks involved in the stress evaluation process. This framework follows a commonly used classification of risk [9]: work content and work context. The choice of specific data analysis will depend on the type of instrument used and accordingly on the type of data collected.

**Table 1:** Variables measured in the St.A.R.T. method

**Category**	**Risk**	**OIS**	**Focus group**	**Questionnaire**	**Observations**
Organisational structure		Distribution of gender in the organisation	-	-	-
		Types of contract			
Sentinel events		Sickness absences	-	Intention to quit	-
		Medical examinations			
		Disciplinary sanctions			
		Turnover			
		Injuries			
		Occupational diseases			
Work content	Work environment and work equipment	Existence of environmental risks	Work with adequate equipment	Adequacy and equipment properly functioning	Description of work environment and work equipment
	Task design	Existence of an updated job description	Task clarity	Boredom	Frequency and description of tasks
			Monotony		
			Hitches		Frequency of hitches
		Easy availability of job description to all workers	Interruptions		Frequency of interruptions
	Workload / Work pace	Rating regarding different homogenous families of workers	Rests	Workload	Rests
			Achievable goals	Job demands	
			Adequate time		
	Work schedule	Work schedule	Work schedule	Work during rest days	-
		Shifts	Work during rest days	Hours worked	
		Overwork			
		Unused leave			
Work context	Organisational culture and function	Existence of communication systems	Organisational communication and communication concerning organisational changes	Communication	-
		Existence of benefits			
		Organisational changes occurred			
	Role in organisation	Existence of an organogram	Role clarity	Role clarity	-
			Role conflict	Role conflict	
		Existence of a role description			
	Career Development	Data regarding training activities	Training	Personal development	-
			Career plan		
		Existence of a well-defined career plan	Reward system		
			Performance evaluation system		
		Existence of a well-defined reward system			
		Existence of a well-defined performance evaluation system			
	Decision latitude / control	Rating regarding different homogenous families of workers	Autonomous decision	Control	-
			Autonomy and control in planning work activities		
	Interpersonal relationships at work	Existence of interpersonal conflict management system	Count on colleagues/supervisor/organisation help	Social support from colleagues/supervisor/organisation	Interactions with colleagues/supervisor and customers
		Existence of technical/personal support system	Mutual respect Conflict	Conflict	
				Leadership	
	Home-work interface	Benefit	Work-family conflict	Work-family conflict	-
		Existence of policies that facilitate the work-family balance		Family work facilitation	
Individual factors	Independent or moderating variables	-	-	Personality traits, self-efficacy	-
	Outcome variables	-	-	Job burnout, work engagement, health symptoms, sickness absence	-

The table provides examples about variables that will be investigated.

Looking at the table we can find categories and types of risk (horizontally) and the methodologies used (vertically). The cells provide some examples of constructs measured.

Analyzing data in mixed methods research is a complex step. Respectively, triangulation design Creswell and Plano Clark [53] indicate concurrent data analysis that tries primarily to conduct a separate initial data analysis for each of the databases: this step is followed by merging the datasets so that a complete picture is developed from all datasets. For the merging procedures two tecniques are usually used: data transformation or comparison with a matrix or discussion (that is a frequently used approach).

Taking the example of the “Task Design” risk, we can evaluate it in an integrated way. The OIS proposes to the experts of the steering committee some questions regarding task design, such as whether there is an updated job description and whether it is easily available to all workers. The focus group conducted by a trained psychologist will refine the data gathered by the steering committee and collect qualitative data regarding the clear definition and the equal distribution of job tasks between workers, the balance between workers’ skills and job demands and the task variety. It is possible to collect data regarding subjective perception with a validated scale (e.g., boredom [57]). Therefore we can also obtain objective data with observations made by trained psychologists that rate and measure the different tasks that workers do, and the hitches and the interruptions occurred. In this way it is thus possible to collect a large amount of data.

The observation instrument is useful only for some risk categories such as workload/work pace, and it is unusable for measuring other variables such as role in the organisation.

The questionnaire also enables the researcher to collect data regarding the personal factors that can have a direct effect or moderate the impact on workers’ health.

In a previous study [49] conducted in a retail context, the integration between objective and subjective measures has already been tested, and the dimensions related to work activities were satisfactorily correlated. The data showed a relation between the relationship with customers (subjectively measured with questionnaires) and the customer queue (objectively measured with an OC). On the other hand, workload and boredom (measured with subjective and objective methods) showed a discrepancy. Beyond the validation of the instruments and this first research on integration of quantitative data, the aim of this work is more ambitious: to validate a method of stress evaluation integrating the different data collected using mixed methods research.

Hence this method allows analysis of data between groups and the relations between the different psychosocial variables.

In particular, whereas the main level of analysis is the presence of psychosocial factors in the organizational contexts, the aim of this protocol is not the evaluation of to what extent every worker feels stressed, but to what extent psychosocial risk factors have as an impact on the workers in a specific work context and what the relationships are between them and the perceptions of stress and reduced well-being of workers. Every worker is considered representative of the foresaid above attached to the fact that psychosocial factors have to be evaluated respect to the groups of workers composed on different work activities and stress risk exposures.

In regard to the discrepancies that could occur (in our data, no more than 10%), we strictly adhere to the general framework, where the steering committee is the first instance as for competency, vision and data interpretation. So, significant discrepancies should be (and have actually been) reported to the steering committee.

## Discussion

A change in Italian legislation on occupational health and safety (Law 81/2008) occurred, and a more compelling and detailed obligation to monitor psychosocial risk factors has been introduced, even if among scientists and professionals in the field there is not yet a full agreement regarding their definition and measurement.

Considering such a situation, it is necessary to find valid, reliable and practical tools to assess psychosocial risk factors at work, which we have operationalised as job demands and resources, in order to prevent the former and promote the latter.

Since scientific research and professional practice show that stressful conditions do not automatically lead to stress, which also depends on personal and resource characteristics, it is important to rely on different typologies of data collection to evaluate correctly work-related stress. The StART method follows the lead of several authors recommending the use of innovative methodologies and a multi-method approach (e.g., [36,58]). This kind of methodology is increasingly used in the health and safety field (e.g., [59]).

Based on these premises, the study protocol allows: 

a) positive and negative aspects of work to be assessed conjointly, using an approach that considers simultaneously at the same time job demands and job resources (e.g., [10-12]), which has been proven to be able to explain negative and positive outcomes of work demands (e.g., health, job satisfaction). Assessing job resources is important for two main reasons. First, researchers have pointed out the stress-buffering effect of job resources, indicating that high job demands will result in job strain unless workers have sufficient job resources to deal with their demanding job (e.g., [22,23,60]. Secondly, in line with current regulations, job resources constitute a protective factor for potential critical situations in the work environment and act as a starting point to formulate corrective measures. In other words, in a risk assessment process aimed at reducing and managing criticality, in addition to identifying harmful risk factors that negatively influence health and well-being (i.e., workload, conflict, etc.), it is also useful to detect the resources available in the work context (i.e., protective factors) [45].

b) the validity of psychosocial risk measurement to be increased, overcoming the limitations previously cited, by assessing work-related stressors using different kinds of data: i) organisational archival data (OIS), which could also be compared with benchmarking data; ii) qualitative data (focus group); iii) worker perception (questionnaire); and iv) observational data (OC). Such integration of data can reduce theoretical and methodological bias typical of stress research in a work setting (e.g., common method variance), and allows researchers and professionals to obtain a more reliable description compared with the use of a single analysis tool, in that it provides a more articulated vision of psychosocial risks.

There is still debate among researchers and practitioners about the best combination of different types of data in risk assessment. Indeed, no matter what the preferred methodological orientation, it is challenging to address both organisational needs and normative obligations, finding an appropriate response that couples measurement issues (validity and reliability) with usability (i.e., utility, cost-effectiveness).

In recent years, many researchers have developed methods in an attempt to overcome the limitations (e.g., [25,61]). The ways in which our method addresses the methodological issues and allows for an improvement in risk assessment and management are twofold.

First, following the suggestion made by Kompier [26], who argued that a multi-source approach may overcome the dichotomy between subjective and objective measures, our method includes objective (i.e., archival data, observational data) and subjective (self-report questionnaires, focus groups) data, in order to reduce the measurement error typical of each kind of data-gathering tool. The second specific feature of our method concerns the research design. As stated by Kelle [29], both quantitative and qualitative methods have specific limitations as well as particular strengths. Thus, it is proposed to mix them to compensate for their mutual weaknesses. Our method uses mixed methods research to combine quantitative and qualitative research techniques, methods, approaches, concepts or languages into a single study. The protocol presented adds ulterior information on how to evaluate psychosocial risks at work, and, to the best of our knowledge, no previous study has used a mixed method design in risk assessment for work-related stress.

Besides these two aspects, it is worthwhile noting that the cyclic process (starting from the involvement of top management representatives in the discussion of results) is an operational response to the needs of those organisations that intend to use the results of the assessment process to improve human resource practices and procedures. In fact, results can be used to implement corrective measures to reduce the influence of psychosocial risks and to design the approach to risk management in the firm responding to the need for usability. Furthermore, the implementation of the method ensures in the long term two aspects: a primary prevention for psychosocial risk management in that it aims to reduce or modify the intensity, frequency and or duration of organisational demands [14], and a large amount of data (also longitudinal).

Some limitations of this study protocol that can help the researcher to apply it have to be mentioned. First of all, the full application of the method requires the top management to be highly engaged in the process. This involvement may be easier in organisations with participatory climate and culture, while it may be more difficult to obtain in other contexts, e.g., individualistic ones.

A second limitation of the method is the duration and complexity of its application. While such limitation is shared with almost every other method based on control cycle and workers’ involvement, the implementation has to be carefully planned and monitored to avoid time and resources loss. It is, however, to be acknowledged that this limitation also represents a strength and a novelty characteristic of this method.

Lastly, while flexibility is one of the strengths of the method, it could also be seen as a weakness, since it may limit comparability of data among different firms. However, in the long term the development of a database of enterprises pertaining to different sectors will allow for normative values to be set to assess standards of reference. Furthermore, it allows a large amount of data to be collected.

Future studies will need to provide a more complete picture of how different measures contribute to a valid risk assessment of work-related stress. Based on theory and empirical data, it is clear that both overlaps and unique contributions exist for observational, organisational and perceptual measures. However, a clear-cut solution about the best (i.e., the most reliable and valid) combination of such measures seems quite far off.

## Abbreviations

StART: Stress assessment and research toolkit; OIS: Organisational indicators sheet; OC: Observational checklist.

## Competing interests

The authors declare that they have no competing interests.

## Authors’ contributions

All the authors discuss the methodological biases of the study protocol providing an important contribution. Besides this, all the authors discussed the structure and contents of the study protocol. MCT, RB and FSV prepared a first draft of the manuscript that it has been discussed thereafter by all the authors. IB, MV and SS prepared a second draft of the manuscript and then after a discussion of all the authors MD and DG provide the ultimate version of the manuscript. All authors read and approved the final manuscript.

## Authors’ information

The authors belong to research teams operating for a long time within the field of occupational health, with a special attention towards the knowledge transfer from academic research to professional interventions.
